# Preclinical studies of Apogossypolone: a new nonpeptidic pan small-molecule inhibitor of Bcl-2, Bcl-X_L _and Mcl-1 proteins in Follicular Small Cleaved Cell Lymphoma model

**DOI:** 10.1186/1476-4598-7-20

**Published:** 2008-02-14

**Authors:** Alan A Arnold, Amro Aboukameel, Jianyong Chen, Dajun Yang, Shaomeng Wang, Ayad Al-Katib, Ramzi M Mohammad

**Affiliations:** 1 Department of Internal Medicine, Division of Hematology/Oncology, Wayne State University School of Medicine, Detroit, Michigan, USA; 2 Department of Internal Medicine and Medicinal Chemistry, University of Michigan Comprehensive Cancer Center, Ann Arbor, Michigan, USA; 3 Ascenta Therapeutics, Inc., Malvern, Pennsylvania, USA

## Abstract

Elevated expression of anti-apoptotic Bcl-2 family proteins have been linked to a poor survival rate of patients with Follicular Lymphoma (FL). This prompted us to evaluate a very potent non-peptidic Small-Molecule Inhibitor (SMI) targeting Bcl-2 family proteins, Apogossypolone (ApoG2) using follicular small cleaved cell lymphoma cell line (WSU-FSCCL) and cell isolated from lymphoma patients. ApoG2 inhibited the growth of WSU-FSCCL significantly with a 50% growth inhibition of cells (IC_50_) of 109 nM and decreased cell number of fresh lymphoma cells. ApoG2 activated caspases-9, -3, and -8, and the cleavage of Poly (ADP-ribose) polymerase (PARP) and Apoptosis Inducing Factor (AIF). In the WSU-FSCCL-SCID xenograft model, ApoG2 showed a significant anti-lymphoma effect, with %ILS of 84% in the intravenous and 63% in intraperitoneal treated mice. These studies suggest that ApoG2 can be an effective therapeutic agent against FL.

## Introduction

Follicular Lymphoma (FL) is fifth leading diagnosed cancer estimated with over 63,000 new patients in 2007 within the United States. FL is the most common type of low grade lymphoma and the second most common subtype of lymphoma worldwide. The natural history of FL has not changed over the last 3 decades with median survival ranging from 7–10 years; the disease is considered incurable using various anti-cancer agents [1-3]. Current treatment strategies are aimed at producing remissions, preserving vital organ function and enhancing patients' quality of life [4]. Phase II trials of CHOP followed by Tositumomab/Iodine I-131 demonstrated progression free survival of 67% of patients [5]. Phase III trials of Rituximab shows improved progression free survival in relapsed/resistant FL and enhanced remission induction when used with CHOP [6], with these improvements in the treatment, to date there is not a cure except for a limited number of patients who present with localized disease. Therefore, developing targeted therapy to proteins such as Bcl-2 that prevent death of lymphoma cells is advantageous.

Bcl-2 plays an important role in the lymphomagenesis of FL. Bcl-2 was originally discovered in FL as a proto-oncogene involved in the t(14;18) chromosomal translocation [7-9]. This genetic event is found in more than 85% of FL. It has been shown that transfection of Bcl-2 into B-cell lines could increase cell viability and decrease apoptosis of lymphoma cells and additionally, confers resistance of these cells to chemotherapeutic drugs [10]. Thus, interfering with Bcl-2 function is hypothesized to lead to apoptosis of lymphoma cells. Therefore, Bcl-2 is a rational therapeutic target because of its role in regulating the apoptotic pathway.

Structural analysis of the binding clefts in Bcl-2 and Bcl-X_L _using X-ray crystallography and NMR spectroscopy showed conserved similarity in the BH1, BH2, and BH3 domains. These domains create a hydrophobic surface pocket that may represent a binding site for pro-apoptotic members of the Bcl-2 family, such as Bax. The heterodimerization of Bcl-2 family proteins is believed to be pivotal to the anti-apoptotic function of these proteins. Furthermore, site-specific mutagenesis of BH1 and BH2 domains completely abrogrates the anti-apoptotic activity of these proteins [11-13]. These studies suggest that this region could be a promising target for the use of SMIs to induce apoptosis.

Previous studies in this lab using the SMI (-)-gossypol has shown significant growth inhibition *in vitro *and tumor growth inhibition *in vivo *in a diffuse large cell lymphoma model [14]. With a structural based screening approach, TW-37 a more potent SMI to Bcl-2, was discovered [15]. Subsequently, we have confirmed that TW-37 has anti-lymphoma properties in our diffuse large cell lymphoma model [16]. More recently, we developed a new non-peptidic SMI, ApoG2, which binds like the previous SMIs but with a considerably lower K_i_. ApoG2 is a derivative of (-)-gossypol that binds to the Bcl-2 family of proteins in the low nanomolar range with a K_i _of 35 and 25 nmol/L for Bcl-2 and Mcl-1, respectively and a K_i _of 660 nmol/L for Bcl-X_L _[17]. Therefore, the new SMI, ApoG2, could in theory inhibit the anti-apoptotic function of Bcl-2, Bcl-X_L _and Mcl-1 more efficiently and induce apoptosis in FL cells. In this study, we evaluated the effect of ApoG2 on growth of malignant lymphoid cells *in vitro*, its ability to induce apoptosis as well as its anti-lymphoma activity *in vivo *using a SCID mouse xenograft model of FSCCL.

## Materials and methods

### Cell Culture and Reagents

The origin of human FL B cell line WSU-FSCCL was described previously [18]. The cell line was maintained in RPMI-1640 medium containing 10% heat-inactivated fetal bovine serum (FBS), 1% L-glutamine, 100 U/ml penicillin G and 100 μg/ml streptomycin. Cells were incubated at 37°C in a humidified incubator with 5% CO_2_. Fresh samples from patients with pre-B-acute lymphoblastic leukemia (Pre-B-ALL), mantle cell lymphoma (MCL), marginal zone lymphoma (MZL), and chronic lymphocytic leukemia (CLL) were isolated using Lymphoprep (Axis-Shield, Oslo, Norway). ApoG2 was synthesized by modifying (-)-gossypol's two aldehyde groups and prepared at a stock concentration of 1 mM.

### Western Blot Analysis

Proteins obtained from extracts were resolved using 12% SDS-PAGE and transferred to Hybond C-extra membranes (Amersham Life Science, Arlington Heights, IL). Membranes were blocked with 5% milk in Tris Buffer Saline containing 0.05% Tween 20 (TBST) for 1 h at 25°C and then incubated with unlabeled primary antibodies in 2% Bovine Serum Albumin in TBST (1:1000 dilutions in BSA-TBST) overnight at 4°C. Following incubation, membranes were washed in TBST and incubated with corresponding horseradish peroxidase-conjugated secondary antibody (Santa Cruz Biotechnology, Santa Cruz, CA; 1:5000 dilution in 5% milk-TBST) for 1 h at 25°C and then washed before proteins were visualized using an ECL assay (Amersham Pharmacia Biotech, Inc., Piscataway, NJ). Primary antibodies specific for Bcl-2, Bcl-X_L_, Bax, and Mcl-1(Santa Cruz Biotechnology, Santa Cruz, CA) were used. Primary antibodies specific for caspase-3, -9, PARP and AIF were obtained from Cell Signaling, (Danvers, MA). Protein concentrations were determined using the Micro BCA protein assay (Pierce Chemical Company, Rockford, IL). Quantitation of bands: Values are fold increase of intensity over control based on percentage Integrated Density Value (IDV), using AlphaEaseFC (San Leandro, CA).

### Detection of apoptosis

ApoG2 effectiveness to induce apoptosis was quantified using two DNA intercalating dyes. 3,6-bis [Dimethylamino] acridinium chloride hemi-[zinc chloride] (termed "acridine orange" or AO) and 3,8-diamino-5-ethyl-6-phenyl-phenanthridine bromide (termed "ethidium bromide" or "EB") were used (Invitrogen, Carlsbad, California). AO stains DNA bright green allowing visualization of the nuclear chromatin pattern. EB stains DNA orange but is excluded by viable cells. Dual staining allows separate enumeration of populations of viable-non-apoptotic, early-apoptotic, late-apoptotic and necrotic cells. The assay was performed by combining 100 μg/μl of AO and 100 μg/μl of EB in PBS. WSU-FSCCL cells and primary lymphocytes from patients were incubated for the indicated times and concentrations, centrifuged at 2000 g for 5 m at 4°C, resuspended in 50 μl of PBS, with a resultant of 20 μl of suspension being counted using a Nikon Fluorescent microscope. For each sample at least 200 cells were counted.

### Flow cytometric analysis of apoptosis

Apoptosis was determined by the flow cytometric measurement of phosphatidylserine exposure using Annexin V FITC and Propidium Iodide stain. Cells were grown in the presence or absence of ApoG2 and then centrifuged at 2000 × g for 5 min. The cells were then resuspended in PBS and stained with fluorescent conjugates of Annexin V (BioVision, Mountain View, CA) for 1 hour and propidium iodide for 30 min, and then analyzed on a FACScan machine (BD, San Jose, CA).

### Detection of Caspase Activity

WSU-FSCCL cells exposed to 0.35 and 3.50 μM ApoG2 for 0 to 72 hrs were incubated on ice for 30 minutes in cell lysis buffer (Sigma-Aldrich, St. Louis, MO). The supernatant after centrifugation at 14,000 g at 4°C was collected and proteins were quantified according to the bicinchoninic acid protein assay methodology (Pierce Chemical, Rockford, IL.). A total of 50 μg protein in a volume of 50 μl cell lysis mixture was resuspended on ice as triplicates in a 96-well plate; 50 μl of 2× reaction buffer containing 10 mM DTT was added to each sample (MBL International, Woburn, MA); 50 μM final concentration substrates for caspase-3 (DEVD-pNA) and caspase-9 (LEHD-pNA; MBL International, Woburn, MA) were added to each sample for a total volume of 100 μl and incubated for 180 m at 37°C. Free pNA released from the labeled synthetic substrate on cleavage by active caspase was measured on a fluorescence plate reader (Molecular Devices, Sunnyvale, CA) at 405 nm.

### SCID/Human Xenograft

Female ICR SCID mice were obtained from Taconic Laboratories (Germantown, NY) and were housed and treated in the Wayne State University School of Medicine under an approved protocol. Four week old mice were injected intraperitoneally (i.p.) with 5 × 10^6 ^WSU-FSCCL cells. ApoG2 was injected 25 mg/kg QD × 5 days either IP or intravenously (i.v.). Mice were observed daily and euthanized when they appeared ill. Animals' activity, weight and survival were monitored three times a week; mice were sacrificed when they developed hind region paralysis, had decreased activity and weight loss of 15% or more, or death was felt to be imminent. Necropsy was carried out and the extent of macroscopic disease was identified with all major organs being taken for microscopic pathological examination. Major organs included the brain, femur (for bone marrow), heart, kidney, liver, lungs, pancreas, retroperitoneal fat, and spleen. Peripheral blood smears were examined for evidence of circulating lymphoma cells.

Survival curves were created using the product limit of Kaplan and Meier, and compared using the log-rank test. The end point for assessing anti-lymphoma activity was calculated by percent increase in host life span (%ILS). %ILS = 100 × MDD (median day of death of the treated tumor-bearing mice) – (MDD of tumor-bearing control mice)/MDD of the tumor bearing control mice.

### Statistical analysis

Apoptosis induction by AnnexinV/PI stains and AO/EB were compared to control by the student t-test. Survival functions were estimated using the Kaplan-Meier method and compared by the log-rank test. P-values < 0.05 were considered statistically significant. All statistical analyses were evaluated using GraphPad Prism 4 (San Diego, CA)

## Results

### Effect of ApoG2 on WSU-FSCCL Cells

**Figure 1:**
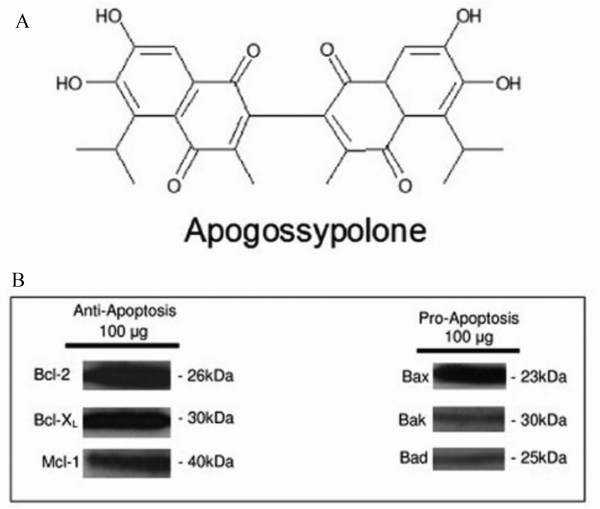
**The chemical structure of ApoG2; an analogue of (-)-gossypol (A).** Baseline endogenous expression of anti-apoptotic (Bcl-2, Bcl-X_L _and Mcl-1) and pro-apoptotic (Bax, Bak, and Bad) proteins in WSU-FSCCL cell line (B). Protein obtained from cell lysates (100 μg) of WSU-FSCCL cells were separated on a 12% SDS-PAGE. Proteins were immunoblotted using specific primary antibodies to Bcl-2, Bcl-X_L_, Mcl-1, Bax, Bak, and Bad. Primary antibodies were diluted in 2% BSA. Secondary HRP conjugates were diluted in 5% milk.

**Figure 2:**
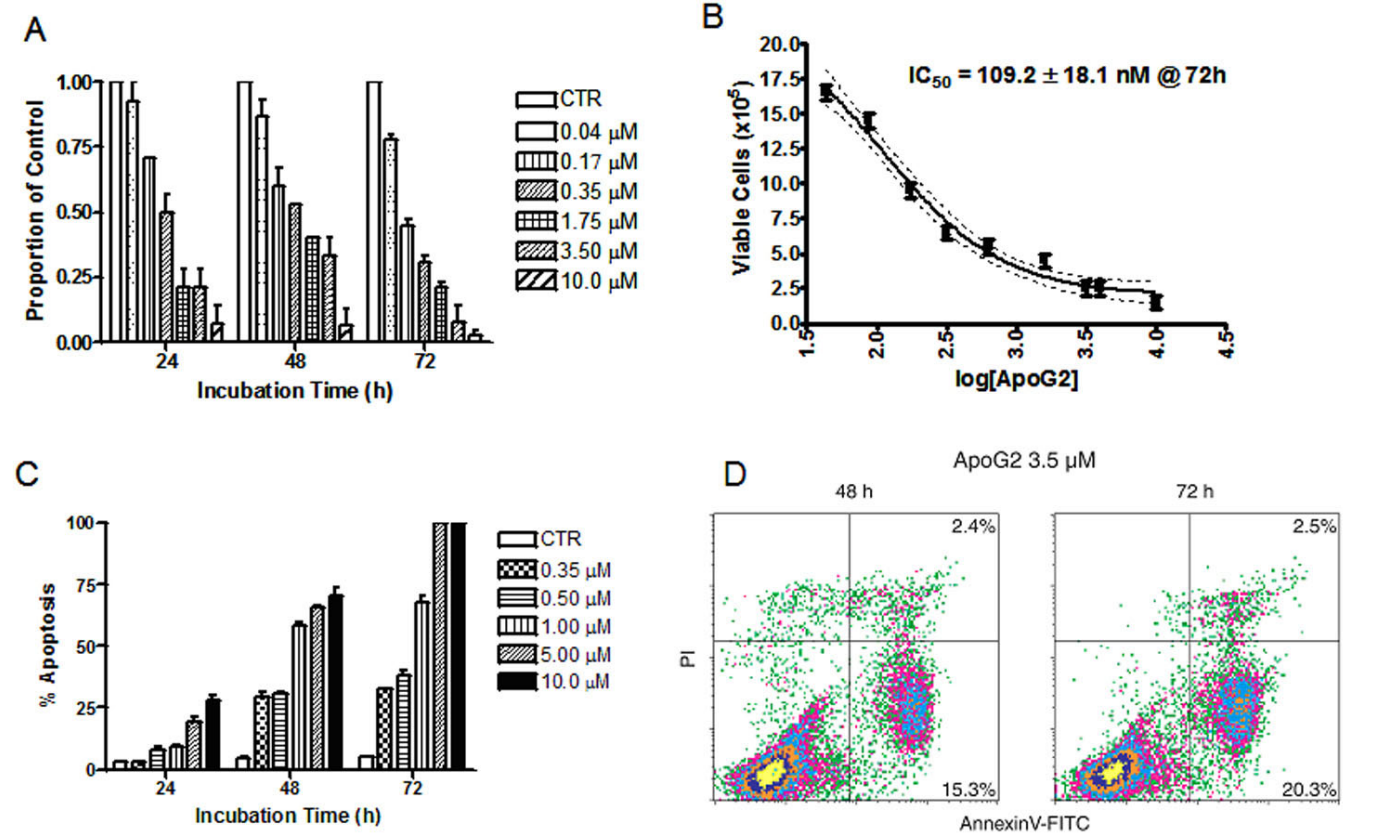
**Growth inhibition, IC_50 _and apoptosis in WSU-FSCCL cells expose to ApoG2**. WSU-FSCCL cells were seeded in 24 well culture plates at a density of 2 × 10^5 ^cells per 1 mL of RPMI 1640 + 10% FBS. ApoG2 was added at 0.04 μM to 10.0 μM concentrations and plates were incubated at 37°C in CO_2 _incubator for 24 to 72 hours. Trypan blue exclusion dye was used to determine viable cells (A). Fifty percent cell inhibition of viable cells was calculated from trypan blue exclusion assay, IC_50 _= 109.2 ± 18.1 nM at 72 h (B). AO/EB was quantitated by counting several fields of cells on frosted slides (C). Counting was performed using Nikon light and fluorescent microscope. AnnexinV stain was used to confirm percentage of apoptotic cells incubated for 48 and 72 h (D).

The structure of ApoG2 is shown in figure 1A. To study if ApoG2 is effective in our FL cell line, WSU-FSCCL, we determined the baseline expression levels of anti-apoptotic, Bcl-2, Bcl-X_L _and Mcl-1 and expression levels of pro-apoptotic, Bax, Bak, and Bad proteins (Fig. 1B). Our western blots show that our FL cell line has high expression of anti-apoptotic proteins (Bcl-2, Bcl-X_L _and Mcl-1) and pro-apoptotic protein Bax, but low expression of pro-apoptotic proteins (Bak and Bad). This profile predicts that ApoG2 should be an effective agent in this model. To study cytotoxic effects of ApoG2, WSU-FSCCL cells were exposed to increasing concentrations of the SMI for 24 to 72 h. We exposed WSU-FSCCL cells to ApoG2 at concentrations of 0.04, 0.08, 0.18, 0.35, 0.70, 1.75, 3.50, 5.00, and 10.00 μM. ApoG2 significantly inhibited the growth of WSU-FSCCL in a concentration and time dependent manner (Fig. 2A). For example, ApoG2 at a concentration of 10.00 μM ApoG2 inhibited the growth of WSU-FSCCL cells by 90% at all incubation times. Plotting the log of the ApoG2 concentrations, we calculated an IC_50 _of 109.2 nM at 72 h (Fig. 2B). There was also a time and concentration dependent increase in apoptosis, as enumerated by AO/EB. ApoG2 induced a statistically significant increase in apoptosis over control at 48 and 72 h, with all concentrations; P = 0.0034, 0.0162, 0.0067, 0.0456 and 0.0322. Complete apoptosis was observed with ApoG2 concentration of 5.0 μM and 10 μM 72 h (Fig. 2C). We have confirmed apoptosis by Annexin V/PI staining; statistically significant apoptosis was induced by ApoG2 (P = 0.0324), with 15% and 20% positivity at 48 and 72 h, respectively, compared to control (Fig. 2D).

### Effect of ApoG2 on Primary Fresh Cases

**Figure 3:**
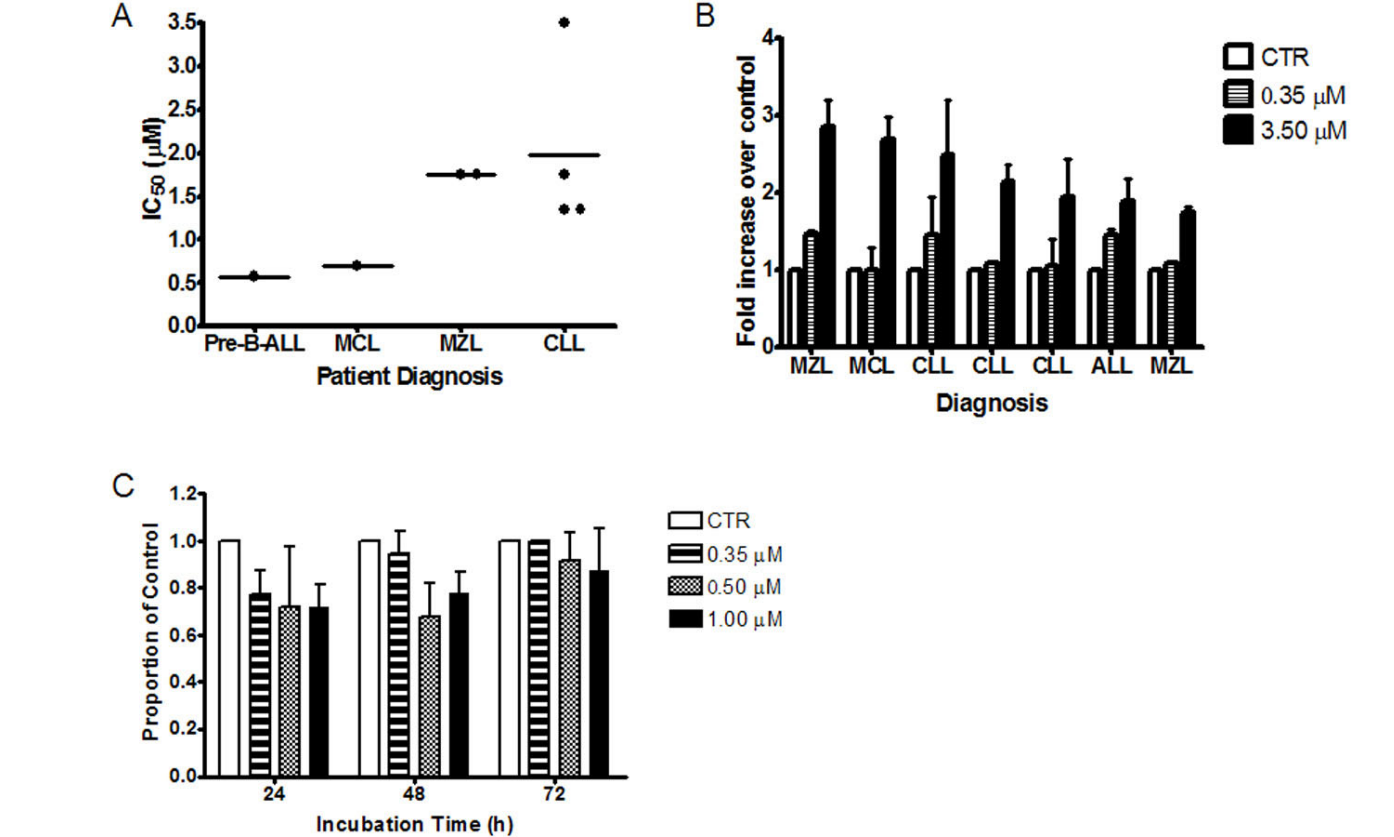
**Cell viability and apoptosis of patient samples, and cell viability of peripheral blood lymphocytes exposed to ApoG2.** Patient samples (A,B) and peripheral blood lymphocytes from a normal donor (C) were seeded in 24 well culture plates at a density of 4 × 10^5 ^cells per 1 mL of RPMI 1640 + 10% FBS. ApoG2 was added at 0.35 μM to 3.50 μM concentrations and plates were incubated at 37°C in CO_2 _incubator for 24 to 72 hours. Trypan blue exclusion dye was used to determine viable cells. Trypan blue exclusion was used to determine viable cells (A and C). Each dot represents a different patient. (-) is the mean of ApoG2 concentration that causes 50 percent growth inhibition at 72 h, Pre-B-ALL represents 24 h only (A). AO/EB was quantitated by counting fields of cells on frosted slides (B).

The IC_50 _of ApoG2 at 72 h was determined on MCL, MZL, and CLL fresh patient samples. In general, they fall into a susceptible group of Pre-B-ALL and MCL or a less susceptible group of MZL and CLL. Pre-B-ALL sample showed an IC_50 _of 0.50 μM at 24 h. The MCL sample showed an IC_50 _of 0.70 μM. MZL patient samples were more resistant and showed an IC_50 _of 1.75 μM. CLL patient samples were resistant to ApoG2 having a range of IC_50 _from 1.35 to 3.50 μM (Fig. 3A). ApoG2 induced at least a 1.5-fold increase in apoptosis over control with concentration of 3.5 μM (Fig. 3B). Conversely, exposure of normal peripheral blood lymphocytes to 0.35, 0.50 and 1.00 μM ApoG2 did not show any statistically significant cell death at 72 h (Fig. 3C).

### Effect of ApoG2 on the activation of Caspases in WSU-FSCCL cells

**Figure 4:**
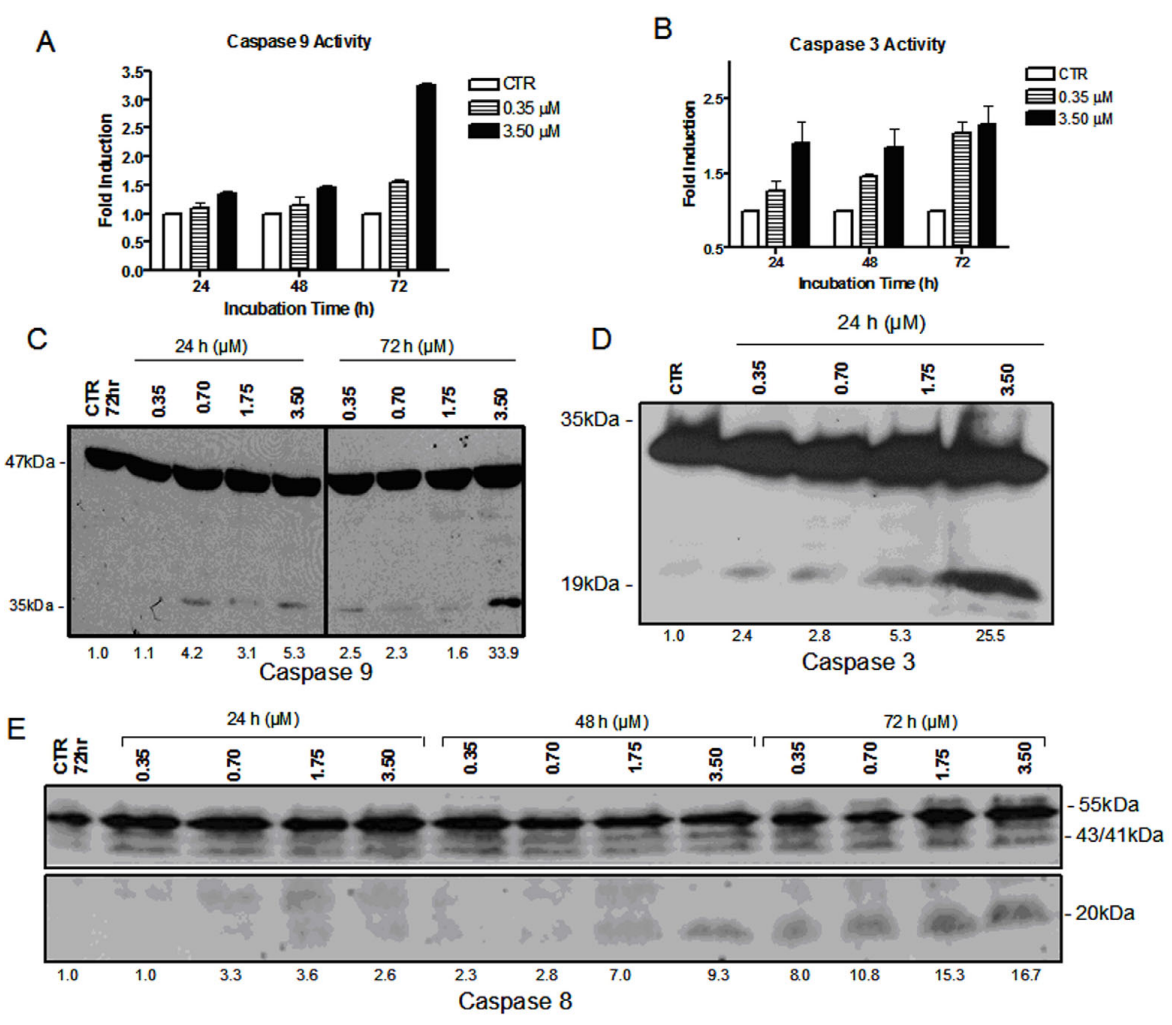
**Induction of caspase activation and caspase cleavage in WSU-FSCCL cells expose to ApoG2.** Caspase-9 and -3 colorimetric activity assay on WSU-FSCCL cells exposed to ApoG2 at indicated times and concentrations. 50 μg of protein from cell lysates were incubated in triplicate with the corresponding substrates for caspase-9 (LEHD-pNA) caspase-3 (DEVD-pNA). Free pNA is released from the labeled synthetic substrate on cleavage by active caspase and analyzed (A and B). Protein obtained from cell lysates (100 μg) of WSU-FSCCL were separated on a 12% SDS-PAGE. Proteins were immunoblotted using specific antibodies to caspase-9, -3, and -8. WSU-FSCCL cells exposed to ApoG2 at indicated times and concentrations (0.35 μM to 3.50 μM) (C, D, and E). Primary antibodies were diluted in 2% BSA. Secondary HRP conjugates were diluted in 5% milk. Quantitation of bands: Values are fold increase of intensity over control based on percentage Integrated Density Value (IDV).

WSU-FSCCL was exposed to ApoG2 at 0.35 and 3.50 μM concentrations. ApoG2 at 3.50 μM showed a 3-fold increase in the activation of caspase-9 at 72 hrs (Fig. 4A). ApoG2 at 3.50 μM demonstrated a 2-fold increase of caspase-3 activation for all incubation periods (Fig. 4B). To determine if caspase cleavage in WSU-FSCCL occurred, cells were exposed to ApoG2 at 0.35, 0.70, 1.75 and 3.50 μM and incubated at indicated times. Greater than 3-fold increase of caspase-9 cleavage was detected at concentrations greater than 0.70 μM with 24 h incubation time. Thirty-three fold induction over control of caspase-9 cleavage band was shown at 3.50 μM at 72 h (Fig. 4C). The next downstream protein of the caspase cascade is caspase-3. ApoG2 induced caspase-3 cleavage of 2-fold with concentrations of 0.35 μM and a more intense cleavage band indicating a 25-fold increase was detected at 3.5 μM at 24 h (Fig. 4D). Previous studies have suggested that caspase-3 is also capable of eliciting cleavage and activation of the upstream initiator caspase-8, which may potentiate a feedback amplification loop with further activation of other death substrates. Caspase-8 cleavage was shown to have a 9-fold increase over control at 3.50 μM with 48 h incubation and 8-fold or higher induction at concentrations greater than 0.35 μM with a 72 h incubation period (Fig. 4E).

### ApoG2 Induced Activation of PARP and AIF in the Apoptotic Pathway

**Figure 5:**
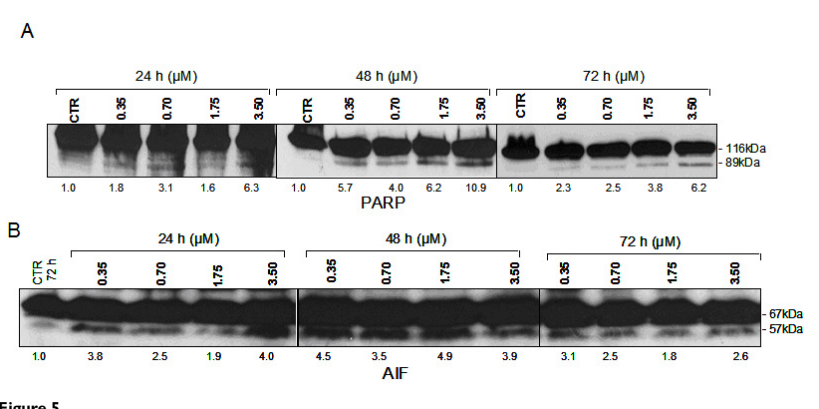
**Induction of PARP and AIF cleavage in WSU-FSCCL cells expose to ApoG2**. WSU-FSCCL cells were exposed to ApoG2 concentrations 0.35 μM to 3.50 μM for 24 to 72 h. Protein obtained from cell lysates (100 μg) of WSU-FSCCL cells were separated on a 12% SDS-PAGE. Proteins were immunoblotted using specific primary antibodies to PARP (A) and AIF (B). Primary antibodies were diluted in 2% BSA. Secondary HRP conjugates were diluted in 5% milk. Quantitation of bands: Values are fold increase of intensity over control based on percentage Integrated Density Value (IDV).

Caspase-3 is primarily responsible for the cleavage of PARP during cell death. WSU-FSCCL was exposed to ApoG2 at 0.35, 0.70, 1.75 and 3.50 μM and incubated for 24, 48 and 72 hrs. As expected, PARP cleavage was shown at all concentrations and incubations periods. Cleaved bands greater than 5-fold over the control were shown at 3.5 μM with all incubation periods (Fig. 5A).

PARP can release apoptosis-inducing factor (AIF), which induces chromatin condensation and large-scale DNA fragmentation when released into the cytosol [19]. Similar to PARP, AIF cleavage was shown at concentrations greater than 0.35 μM. Cleaved bands of AIF greater than 2-fold and (up to 4-fold) were shown at 3.5 μM at 24 hrs (Fig. 5B).

### Determination of Anti-lymphoma Effect of ApoG2 in SCID Mice

**Figure 6:**
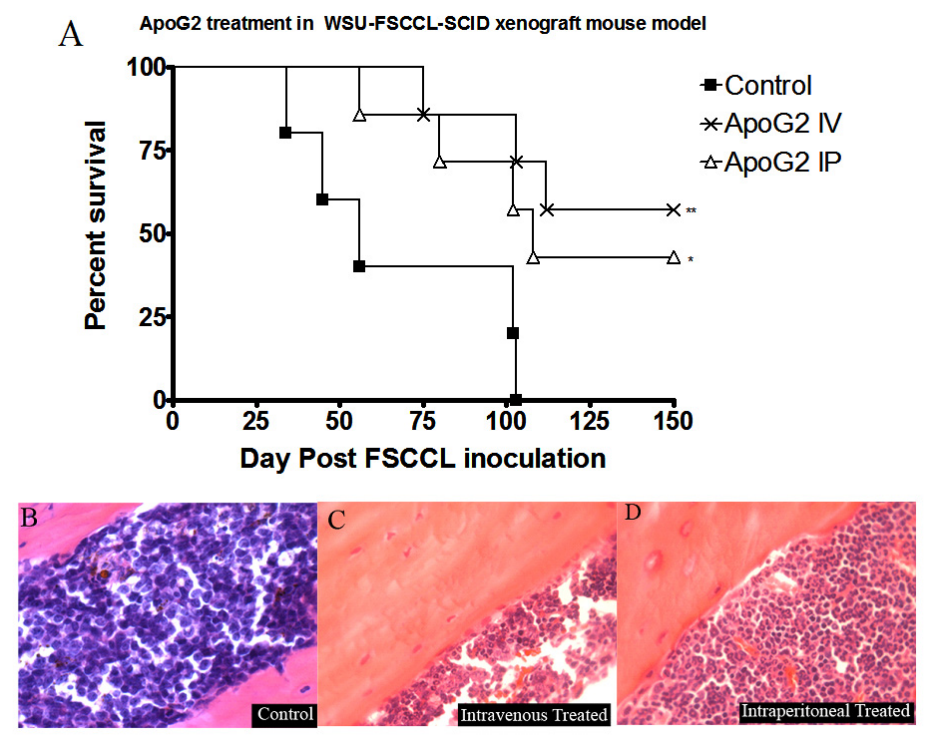
**Survival of WSU-FSCCL-bearing SCID mice according to ApoG2 treatment and H & E stains of bone marrow sections.** WSU-FSCCL cells were injected into the intraperitoneal cavity at 5 × 10^6 ^cells. 25 mg/kg QD × 5 of ApoG2 was injected intravenously or intraperitoneally on day 7 post inoculation. Statistical comparison of survival curves for i.v. treatment and untreated control show a chi square of 8.005 and P = 0.0047. Statistical comparison of survival curves for i.p. treatment and untreated control show a chi square of 4.397 and P = 0.0360 (A). Replacement of the bone marrow with WSU-FSCCL cells in the control mouse (B). Negative bone marrow of intravenous (C) and intraperitoneal (D) treatment with ApoG2.

Previous studies in this laboratory indicated that the MTD for ApoG2 could not be determined. We tested up to 800 mg/kg iv of ApoG2; testing beyond 800 mg/kg were not attempted, due to cost and other logistical issues. For this efficacy trial, 5 × 10^6 ^WSU-FSCCL cells were injected into the intraperitoneal cavity of 7 mice per group. Seven days post WSU-FSCCL inoculation, 25 mg/kg of ApoG2 was injected into each animal either intravenously (i.v.) or intraperitoneally (i.p.) over 5 days. After 105 days, 42% of the i.v.-treated animals, and 60% of the i.p.-treated animals had died from FL (Fig. 6A). Statistical comparison of survival curves for i.v. treatment and untreated control show a chi square of 8.005 and P = 0.0047. Statistical comparison of survival curves for i.p. treatment and untreated control show a chi square of 4.397 and P = 0.0360 (Fig 6A). Pathological evaluation showed that retroperitoneal lymph nodes were diffusely replaced by tumor cells in mice that died (data not shown). The effectiveness of ApoG2 was further demonstrated by bone marrow examination which was completely replaced by tumor cells by day 34 in control animals (Fig. 6B). In contrast, ApoG2 treated mice showed normal bone marrow with no apparent tumor infiltration (Fig. 6C &6D).

## Discussion

FL has been increasing in incidence over the past three decades and is now the fifth most common malignancy in the United States [20]. There are many approaches to the treatment of FL, but the goal of therapy has been to maintain the best quality of life and treat when a patient is at "high risk" or the disease progresses. Standard chemotherapy regimens directed towards these low-grade lymphomas still lack complete curative effects. This may be in part due to the overexpression of Bcl-2, a key molecule of resistance in indolent lymphoma. Overexpression of Bcl-2 has been implicated to play a significant role in the clinical outcome of FL patients [21]. A number of approaches have been sought to target overexpression of Bcl-2 in FL, e.g. downregulation of Bcl-2 protein via antisense oligonucleotides [18,22-24]. Most recently, hydrophobic groove of the Bcl-2 family of anti-apoptotic proteins has become a very attractive target for the design of SMIs. SMIs filling the hydrophobic groove mimic cognate proteins such as Bax and Bid. SMIs directed against BH3 domains have been categorized into at least eight different chemical classifications [25]. Laboratories, including this one, have been in the search to find novel small-molecule inhibitors to Bcl-2 [15,26-29]. Gossypol has been used as an anti-cancer agent against prostate cancer, metastatic adrenal cancer and many other cancers before the BH3 mimetic activity was discovered [30,31]. This discovery has shown that (-)-gossypol, the active enantiomer of gossypol, binds to anti-apoptotic members of the Bcl-2 family, Bcl-2, Bcl-X_L _and Mcl-1, with nanomolar affinities and this active enantiomer has been tested in clinical trails for treatment of patients with advanced malignancies [32].

In clinical trials, gossypol has been associated with side effects, such as emesis and diarrhea, because of the two reactive aldehyde-groups [33]. ApoG2 has been designed and synthesized with the reactive groups completely removed in order to minimize side effects. In addition, ApoG2 has superior stability under both stress and normal conditions compared to (-)-gossypol [34].

It is notable that our study is the first on ApoG2 in FL. The goals of new agents such as ApoG2, are to have higher binding affinity to its targets; ApoG2 has greater than 8-fold binding affinity to Bcl-2 over its predecessors TW-37 and (-)-gossypol [16,17]. In this study, we have shown a potent anti-lymphoma effect on FL. ApoG2 shows an IC_50 _of 9- and 18-fold lower when compared to TW-37 or gossypol. When compared to HA14-1, which is a SMI to Bcl-2 used against leukemia cell lines HL60 and K562 [29], ApoG2 has a IC_50 _which is 200-fold lower. The SMI ABT-737 has a considerably lower IC_50 _(8 and 30 nM) when used against FL cell lines, but ABT-737 does not bind to Mcl-1 and thus Mcl-1 expression could result in resistance. In comparison, ApoG2 targets all these three anti-apoptotic proteins [27,35,36]. In our study, ApoG2 is effective against FL, pre-B-acute lymphoblastic leukemia, mantle cell lymphoma, marginal zone lymphoma, as well as chronic lymphocytic leukemia. Therefore, ApoG2 could potentially be a more effective drug in the lymphoma clinic spanning a greater array of patients.

With the binding of ApoG2 to Bcl-2 family of proteins, it would be expected that ApoG2 would lead to activation of downstream apoptotic proteins. The mechanism of action of ApoG2 has not been elucidated in FL. We show here that ApoG2 can activate the initiator caspase-9, and the effector caspase-3, and induce caspase cleavage in nanomolar concentrations. Moreover, ApoG2 can lead to the activation of caspase-8 which serves as amplification loop together with caspase-3 [37,38]. PARP and AIF have been implicated in the final stages of apoptosis. They play a role in the chromatin condensation and DNA fragmentation. We show that ApoG2 activates PARP and AIF in the nanomolar range.

These findings clearly demonstrate that ApoG2 can activate the Bcl-2 apoptotic pathway in *vitro*. The exact mechanism of action of ApoG2 is unclear. Likely mechanisms are that ApoG2 binds to Bcl-2 (or Mcl-1, Bcl-X_L_, A1, Bcl-w) and prevents its association with BH3-only pro-apoptotic proteins, thus unleashing the pro-apoptotic proteins to participate in the apoptotic response. Work in our laboratory is being done to further elucidate the mechanism of ApoG2 action.

Many agents targeting the Bcl-2 family were shown to have activity in *vitro*. However, the main goal of our research endeavor is to find out if ApoG2 can make its way into clinical trials. Here, we tested the anti-lymphoma activity of ApoG2 in *vivo*. The endpoint for treatment efficacy is survival of the mice bearing the human FL cells. Our study showed that regardless of route of injection (i.p. or i.v.), ApoG2 could significantly increase the life span of lymphoma-bearing SCID mice by at least 42% (Fig. 6A). Moreover, ApoG2 was safe and well tolerated up to 800 mg/kg with no weight lose in all treated animals. ApoG2 has an undetermined MTD, and a large therapeutic window of 25 to 800 mg/kg; with effective dose of only 25 mg/kg; compared to ABT-737, that has a therapeutic window of 25 to 100 mg/kg, with an undetermined MTD [39].

In closing, we have shown that ApoG2 can be a potential novel agent against FL. Our data suggest that ApoG2 also could be used in several different types of lymphoid malignancies. ApoG2 in this study does show efficacy for treatment of FL as a single agent; it can prove to be even more effective when used in combination with standard chemotherapy.

## Abbreviations

ApoG2 = Apogossypolone; SMI = Small-Molecule Inhibitor; IC_50 _= concentration of agent with fifty percent of growth inhibition; PI = propidium iodide; ILS = increase in life span; NHL = Non-Hodgkin's Lymphoma; FL = Follicular Lymphoma; CHOP chemotherapy = C-cyclophosphamide, H-doxorubicin Hydrochloride, O-oncovin, P-prednisone; PARP = Poly (ADP-ribose) polymerase; AIF = Apoptosis Inducing Factor; ALL = acute lymphoblastic leukemia; MCL = mantle cell lymphoma; MZL = marginal zone lymphoma; CLL = chronic lymphocytic leukemia.

## Competing interests

Dajun Yang has competing interests with Ascenta Therapeutics Inc., as Senior Vice President of Research, shareholder and co-founder.

Ascenta Therapeutics Inc., has licensed the technology related to apogossypolone (ApoG2) from the University of Michigan. Both the University of Michigan and Shaomeng Wang own equity in Ascenta and Shaomeng Wang also serves as a consultant for Ascenta.
